# Impressive Short-Term Improvement in Functional Outcome and Quality of Life after Primary Total Hip Arthroplasty (THA) in the Orthogeriatric Patient in a Prospective Monocentric Trial

**DOI:** 10.3390/jcm13092693

**Published:** 2024-05-03

**Authors:** Jan Reinhard, Katrin Michalk, Julia Sabrina Schiegl, Stefano Pagano, Joachim Grifka, Günther Maderbacher, Matthias Meyer, Tobias Kappenschneider

**Affiliations:** Department of Orthopaedic Surgery, University Medical Center Regensburg, 93077 Bad Abbach, Germany; katrin.michalk@ukr.de (K.M.); julia2.goetz@ukr.de (J.S.S.); joachim.grifka@ukr.de (J.G.); guenther.maderbacher@ukr.de (G.M.); matthias.meyer@ukr.de (M.M.); tobias.kappenschneider@ukr.de (T.K.)

**Keywords:** orthogeriatric patients, primary total hip arthroplasty (primary THA), elderly patients, older patients, geriatric patients, frailty, EQ5D-5L, WOMAC, comprehensive geriatric assessment (CGA)

## Abstract

**Background/Objectives**: Osteoarthritis (OA) represents the most frequent chronic joint disease worldwide. Facing an aging population, resulting from the demographic change, the number of primary total hip arthroplasties (THA) will further increase. Although the geriatric patient strongly differs from the younger one, the current literature on elective orthopedic surgery in the geriatric patient is scarce. This work analyses, whether geriatric patients receiving primary THA significantly improve in terms of their (1) mobility and functional outcome and (2) health-related quality of life at four to six weeks as well as three months postoperatively. **Methods**: In a prospective study design, we analyzed 101 geriatric patients with osteoarthritis of the hip receiving primary THA. The study is part of the ongoing “Special Orthopaedic Geriatrics” (SOG) trial, which is funded by the German Federal Joint Committee (GBA). In addition to a preoperative comprehensive geriatric assessment (CGA), the Western Ontario and McMaster Universities Arthritis Index (WOMAC) and the EQ5D-5L were imposed preoperatively (t0), at four to six weeks (t1), and at three months (t2) postoperatively. **Results**: The 101 enrolled patients had a mean age of 78.1 ± 4.9 years. The total WOMAC score and almost all subcategories significantly improved at four to six weeks as well as three months postoperatively in comparison to the preoperative results (*p* < 0.001). The same was observed for the EQ-5D-5L, showing significant improvement in overall health at both time points (*p* < 0.001) and all subcategories (*p* < 0.05). **Conclusions**: This study implies that a geriatric patient benefits as much from elective primary THA as a younger patient. However, the preoperative comprehensive geriatric assessment with screening for risk factors is of utmost importance. Regarding the aging population, a lot of effort is needed to obtain more knowledge about geriatric patients receiving elective orthopedic surgery.

## 1. Purpose

Affecting more than 240 million people, osteoarthritis (OA) represents the most frequent chronic joint disease worldwide [1,2]. Every second person over 65 years of age suffers from OA [3]. The main symptoms are pain and loss of function in the affected joint [1]. To date, there is no curative therapy for OA, and the disease progresses with time [1]. The worsening of symptoms climaxes in immobilization. When conservative therapy is fully utilized, total joint arthroplasty (TJA) represents the gold standard therapy for advanced osteoarthritis [4].

Industrial countries such as the U.S. or Germany are facing a demographic change, which leads to a rapidly aging population [5]. In the U.S., in 2040, predictions expect a 284% increase in primary total hip arthroplasty (THA) in comparison to 2014 [6,7]. Therefore, over the next decades, the number of geriatric patients meeting multimorbidity and advanced osteoarthritis with the need for elective primary THA is expected to continue to rise [8].

However, the geriatric patient strongly differs from the average orthopedic one. This particular patient population often faces multimorbidity, polypharmacy, malnutrition, immobility, osteoporosis, dementia, and other geriatric syndromes [9,10]. The geriatric patient is reported to have increased complications and mortality rates after TJA [11]. For those geriatric patients showing reduced individual reserve capacity and therefore increased risk for adverse peri- and postoperative events, the multidimensional syndrome of frailty has been established [12]. Different studies reported an association between OA and frailty [13,14,15]

Although the number of geriatric patients receiving elective orthopedic surgery grows continuously, this particular patient type has been neglected in past research. In contrast, over the last few years, a lot of research has been performed on geriatric patients suffering from femoral neck fractures [16]. In 2007, *the Lancet* titled primary THA as the operation of the century [4]. A lot of studies showed good to excellent clinical outcomes after primary THA in the non-geriatric patient [17,18]. To evaluate the functional outcome after THA and the health-related quality of life, the Western Ontario and McMaster Universities Arthritis Index (WOMAC) and the EQ5D-5L are most widely used. Both questionnaires are proven to have high reliability and were validated in different studies [17,19,20].

### Aim of the Study

This work analyzes the clinical and functional outcome of geriatric patients receiving elective, primary total-hip arthroplasty and aims to answer the questions: (1) Did the mobility and functional outcome of patients improve four to six weeks as well as three months postoperatively, measured by the WOMAC score? (2) Did the patients’ health-related quality of life improve four to six weeks as well as three months postoperatively, as measured by the EQ5D-5L-score?

## 2. Patients and Methods

### 2.1. Study Design

This research is part of the trial “Special Orthopaedic Geriatrics” (SOG), representing a monocentric, prospective, randomized-controlled interventional study [8]. It was conducted in agreement with the ethical standards of the Declaration of Helsinki (1975). The study was approved by the local ethics committee (file number 20-1680-101). It is registered in the German Clinical Trials Registry (DRKS00024102), as well as in the international registry for clinical studies of the World Health Organization (WHO). The data collection took place between April 2021 and February 2024. The operation as well as the follow-up assessments were conducted at a tertiary referral center and maximum provider for arthroplasty.

### 2.2. Study Population

The major criteria for inclusion were (1) the age of 70 or above in combination with typical geriatric multimorbidity or (2) the age of 80 or above in combination with advanced primary hip osteoarthritis and utilization of the whole conservative therapy. Patients who (1) were younger than 70, (2) had either previous bony surgery or a tumorous disease on the affected hip, or (3) showed symptoms of acute infection were excluded. Participation was voluntary and withdrawal was possible at any time. This definition is in accordance with the European and German definitions of geriatric patients (Bundesarbeitsgemeinschaft der Klinisch-Geriatrischen Einrichtungen e.V., the German Society for Geriatrics, registered association, and the German Society for Gerontology and Geriatrics, registered association). (See http://www.geriatrie-drg.de/public/docs/Abgrenzungskriterien_Geriatrie_V13_16-03-04.pdf, accessed on 1 February 2024).

### 2.3. Comprehensive Geriatric Assessment and Follow-Up

A comprehensive geriatric assessment (CGA) was performed preoperatively (t0). Follow-up was performed four to six weeks (t1) as well as three months postoperatively (t2). The CGA is routinely used worldwide in clinical settings to assess the clinical journey of geriatric patients with various diseases within a period of one to three weeks, e.g., in acute geriatric care (AGC), transferred to subacute rehabilitation (TSR), or inpatient geriatric rehabilitation. In the scientific context, CGA has also been used in studies on hip fractures as well as TJA, predominantly surveying a study period of days, weeks, and a few months [21,22]. Preoperatively, baseline data such as age, sex, weight, height, obesity level (body mass index (BMI)), care level, preexisting medication, and comorbidities were collated. The Charlson Comorbidity Index (CCI) was subsequently calculated. The Barthel index [23], the Lawton and Brody Instrumental Activities of Daily Living (IADL) scale [24], the Short Physical Performance Battery (SPPB) [25], the Fried Frailty Phenotype [26], the Mini Mental State Examination (MMSE) [27], as well as the Geriatric Depression Scale (GDS-15) [28], were used throughout the course of the special geriatric assessment [8]. In addition, patients were screened for malnutrition using the nutritional risk screening (NRS) [29]. Postoperatively, the operated page, the time of surgery, the length of hospitalization, as well as the reasons for prolongated hospitalization (medical, organizational, and health insurance) were collated. Medical reasons for prolongated hospitalization were caused by surgical and non-surgical complications. Reasons for re-hospitalization were surgical complications such as periprosthetic fractures, pathological fractures, periprosthetic joint infection, wound healing disorders, seroma, and hematoma.

The functional outcome was assessed by using the Western Ontario and McMaster Universities Arthritis Index (WOMAC) and the health-related quality of life by the EQ5D-5L at (1) preoperative admission (t0), (2) four to six weeks (t1), and (3) three months postoperatively (t2). The WOMAC score is a widely used and validated tool to evaluate disorders of the lower extremity [25,30]. It consists of 24 questions and three subscales (pain, stiffness, and problems). The Health and Quality of Life Questionnaire (EQ-5D-5L) addresses the patient’s health-related quality of life [19]. On behalf of the senior study population, the questionnaires were always filled out with the help of a study nurse to clarify obscurities and prevent potential dropouts by misunderstanding questions or leaving answers blank. The maximum follow-up of three months was chosen on purpose for this very old, multimorbid geriatric patient population. In this particular cohort, the risk of possible confounders because of comorbidities, newly detected diagnoses, death, and the worsening of preexisting osteoarthritis of other joints is very high in a long-term follow-up. Therefore, the benefit of the index operation can often only be understood to a very limited extent in a long-term follow-up.

### 2.4. Operation and Postoperative Care

Each patient received primary THA using a muscle-sparing modified Watson–Jones approach [31] via an anterolateral mini-incision in the intermuscular plane between the musculus tensor fascia lata and the musculus gluteus medius [31]. This approach preserves the integrity of the posterior capsule and surrounding muscles, preventing posterior dislocation. A collarless, and depending on the bone quality, a cementless or cemented CORAIL^®^ stem (DePuy Synthes, Raynham, MA, USA) was implanted. Furthermore, a press-fit PINNACLE^®^ acetabular cup (DePuy Synthes, Raynham, MA, USA) and an ALTRX^®^ polyethylene liner (DePuy Synthes, Raynham, MA, USA) were used. Depending on allergies, either a metal head (DePuy Synthes, Raynham, MA, USA) or a BIOLOX^®^ delta ceramic head (DePuy Synthes, Raynham, MA, USA) was used. After the operation, patients were immediately allowed to bear their full weight. A pain management concept based on the three-step analgesic ladder, which was established by the World Health Organization (WHO) [32], was used. Depending on allergies and contraindications, patients received Metamizole 500 mg 4×/d as a basic pain medication. Most patients did not receive non-steroidal anti-inflammatory drugs because of existing comorbidities. In addition, patients received oxycodone/naloxone 10/5 mg on the first day postoperatively. Additional pain medication was given, having regard to the subjective patient rating using the numeric rating scale (NRS).

### 2.5. Data Analyses

Nominal variables are noted in absolute and relative frequencies and metric data as the mean ± standard deviation (SD). The Shapiro–Wilk test was used to test for normal distribution. A repeated measures ANOVA with Bonferroni-adjusted post-hoc analysis was performed. Statistical significance was set at *p* < 0.05. Statistical analysis was conducted with SPSS (IBM SPSS Statistics 28, International Business Machines Corporation (IBM), Armonk, New York, NY, USA).

## 3. Results

### 3.1. Study Population and Preoperative Comprehensive Geriatric Assessment

**Figure 1:**
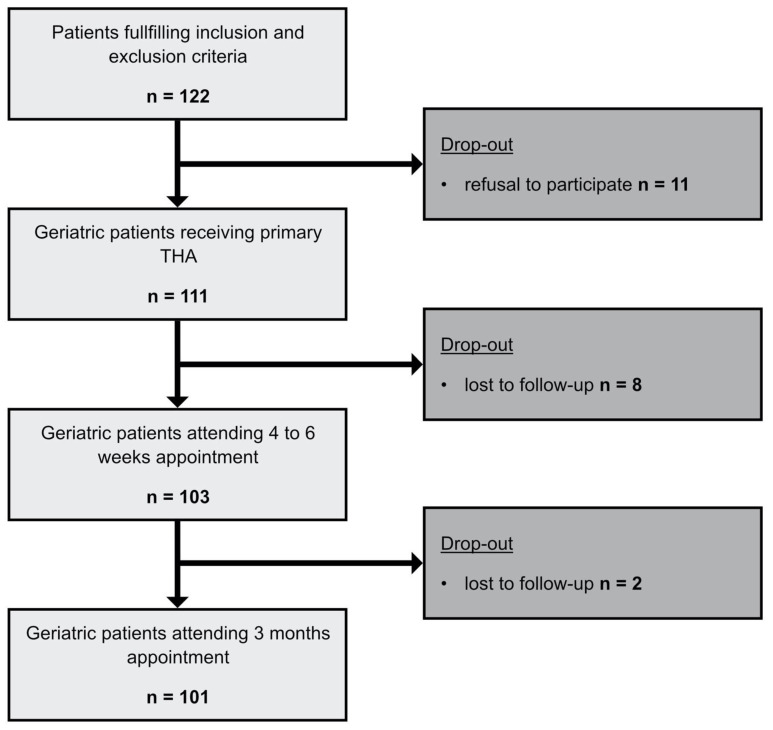
Flowchart methods. Flowchart of the enrollment process and the follow-up. Initial patient population and resulting patients after drop-outs.

During consultation hour, 122 patients that met the inclusion criteria were enrolled. Eleven patients refused to participate and dropped out. Subsequently, 111 patients received the preoperative comprehensive geriatric assessment (CGA) and primary THA. Eight patients were lost to follow-up at the four- to six-week time point (t1) and two patients at the three-month time point (t2). One hundred and one (101) patients were included in the final data analysis (Figure 1).

The study population (n = 101) had a mean age of 78.1 ± 4.9 years. Two-thirds of patients were female. The mean BMI was 28.5 ± 5.1 kg/m^2^. Most patients revealed an obesity level between one and three (Table 1). The preoperative geriatric assessment revealed a mean number of 7.2 ± 3.3 comorbidities and 7.4 ± 4 preexisting pharmaceuticals. The mean calculated Charlson Comorbidity Index (CCI) was 5.4 ± 2. Only five percent of patients showed a risk of malnutrition, according to the Mini Nutritional Risk screening. Evaluation of preoperative Barthel Index, Lawton, and Brody Instrumental Activities of Daily Living Scale (IADL) showed good preoperative scores, demonstrating 93 ± 11.1 and 6.7 ± 1.8, respectively. The Fried Frailty Index revealed a mean of 2.4 ± 1.2, resembling moderate frailty in the study population. Evaluation of the preoperative Mini Mental Status Test (MMST) showed a mean value of 26.9 ± 2.8, resembling normal cognition in most patients. The Geriatric Depression Scale (GDS) indicated no prevalence of depression in the majority of the study population. The Short Physical Performance Battery (SPPB) showed reduced physical function preoperatively (Table 1).

### 3.2. Functional Outcome (WOMAC Score)

**Figure 2:**
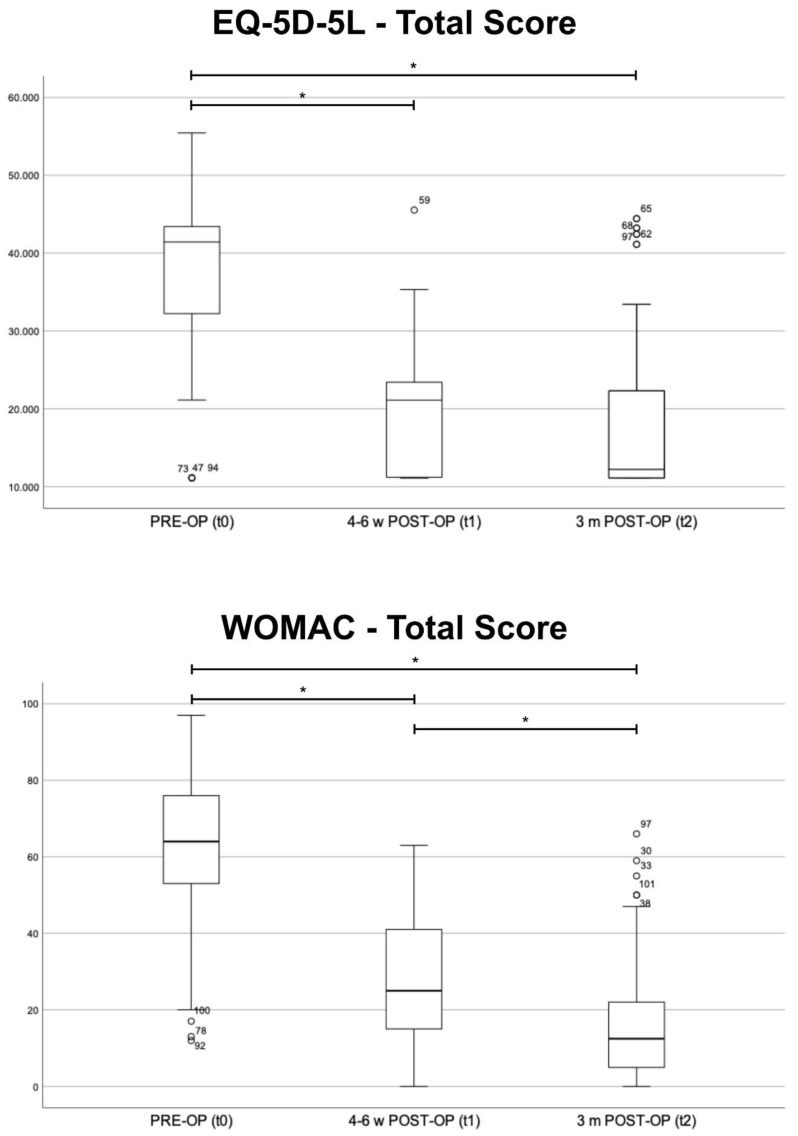
Total results of the EQ-5D-5L and the Western Ontario and McMaster Universities Osteoarthritis Index (WOMAC)—preoperative (PRE-OP, t0), four to six weeks (4–6 w POST-OP, t1) and three months (3 m POST-OP, t2) postoperatively, Boxplot. Significant differences between the three groups are marked by *.

The comparison of the preoperative results (t0) to the results at four to six weeks (t1) and the results at three months (t2), respectively, showed significant improvement in the total score and almost all subcategories (*p* < 0.001). Only the reported need for pain medication did not differ significantly (*p* > 0.05) at four to six weeks postoperatively. The comparison of the results at t1 and at t2 revealed a significantly improved total score (*p* < 0.001) (Figure 2). The comparison of t1 and t2 revealed a significant improvement in almost all subgroups (*p* < 0.05). Only “stiffness during the course of day” and “problems while walking on flat surface” showed an insignificant tendency towards better results (*p* > 0.05) (Table 2).

### 3.3. Health-Related Quality of Life (EQ-5D-5L)

The evaluation of the results of the EQ-5D-5L revealed a significant improvement at both time points (*p* < 0.001) (Figure 2). All sub-scores showed significant improvement (*p* < 0.05). The comparison of t1 and t2 showed a significant improvement relating to general tasks (*p =* 0.01). The other subgroups did not differ significantly (*p* > 0.05) (Table 3).

### 3.4. Secondary Outcome

The operated side was almost equally distributed (50.5% left side). The duration of surgery was, on average, 64.2 ±17.2 min. Patients were hospitalized for a mean of 7.6 ± 2.4 days. In eleven cases, the hospitalization was prolongated for medical reasons, in nine cases for organizational reasons, and in one case because of the requirements of the health insurance, respectively.

## 4. Discussion

To the best of our knowledge, this study is the first to analyze the functional outcome and health-related quality of life after primary total hip arthroplasty (THA) in an orthogeriatric patient in a prospective monocentric trial. We proved a significant improvement at four to six weeks as well as at three months postoperatively in terms of the functional outcome, measured by the WOMAC index, and the health-related quality of life, imposed by the EQ-5D-5L. Both questionnaires are proven to have high reliability and were validated in different studies [19,20]. Clement et al. stated a change of ten points in the total WOMAC score as the minimum clinically important difference. However, they performed the study for patients that received a total knee arthroplasty (TKA) [33]. Our data show a mean difference of 35.5 points between the preoperative and the four- to six-weeks postoperative results, which clearly exceeds the minimum clinically important difference.

The practical implementation showed that many patients needed help completing the questionnaires. This is understandable due to their advanced age and the associated visual, hearing, and writing impairments. Therefore, before surgery, at four to six weeks, and at three months, the orthogeriatric patients were supported by a trained research assistant each time they completed the questionnaires. CGA was performed by an experienced geriatrician [8]. We consider this and the prospective design of the study, with several time points at which the WOMAC and EQ-5D-5L questionnaires were collected on site, to be major strengths of the study. Moreover, this study features a large geriatric patient cohort, meeting a mean age of 78.1 ± 4.9 years, representing an old population, including multimorbidity and frailty. A lot of studies on geriatric patients compromise significantly younger patients.

Over the last few years, a lot of research has been performed on geriatric patients suffering from femoral neck fractures [16,30,34]. In this context, researchers proved an association between the preoperative prognostic nutritional index and postoperative delirium, as well as postoperative mortality [35,36] In contrast, the current literature on elective orthopedic surgery in geriatric patients is scarce. In a retrospective study design, Anderson et al. compared the functional outcome (WOMAC index) and the health-related quality of life (EQ-5D-5L) between three age subgroups of patients, being 60 to 69, 70 to 79, and above 80 years old, respectively [9]. They showed comparable improvements in both scores in all three subgroups, stating that geriatric patients equally improve in comparison to younger patients. The main limitation of this study is the retrospective study design and the fact that no special geriatric assessment was performed. Moreover, the first and only time point for follow-up was set at one year postoperatively; therefore, the results might be biased by other comorbidities that might have occurred over the course of the year. However, this finding is in line with our data, showing significant improvement in terms of functional outcome and health-related quality of life.

On the one hand, older patients benefit significantly clinically from total joint arthroplasty [37]. Kappenschneider et al. showed a clinically meaningful improvement in physical performance in orthogeriatric patients following THA and TKA in only a few weeks [37]. Another recently published study examined the impact of elective total hip replacement surgery on frailty. Pre-frail individuals often regained robustness, and patients with frailty were no longer classified as frail after surgery. Joint replacement was shown to be an effective intervention for improving frailty in patients with symptomatic hip osteoarthritis [12]. In addition to significant improvements in the physical performance and frailty of orthogeriatric patients after THA, we were also able to demonstrate this for the patient reported outcome measures (PROMs). This means that older, multimorbid patients also benefit substantially in terms of self-reported functional outcome and quality of life.

On the other hand, orthogeriatric patients have higher rates of surgical and non-surgical complications, reoperations, hospital readmissions, and blood transfusions [38]. Boniello et al. detected that THAs were associated with an increased risk of complications and mortality in older patients [39]. Meyer et al. described a higher rate of reoperations, hospital readmissions, surgical and non-surgical complications, and blood transfusions in elderly and frail patients [38]. A study by Johnson et al. reported that frailty was associated with increased preoperative complication rates and mortality [40]. The reported higher rates of surgical and non-surgical complications in the orthogeriatric patient should be considered an indication for THA, particularly if geriatric co-management is not possible. Risk stratification is of utmost importance. Preoperative CGA and orthogeriatric co-management can reduce perioperative and postoperative risk by detecting and improving modifiable risk factors [21,38,41,42].

In this aspect, the geriatric patient undergoing elective orthopedic surgery is very different from the geriatric traumatology patient, for whom preoperative optimization is only possible to a limited extent due to time constraints. Harari et al. were able to show, as early as 2007, that precisely these preoperative aspects, in combination with orthogeriatric co-management, can lead to significantly better postoperative outcomes in elective orthopedic surgery [21]. Such patients had significantly fewer postoperative complications, a much better functional outcome, and the hospitalization time could be reduced by several days [21].

Pain medication was administered according to the WHO three-step analgesic ladder. Only as many analgesics as necessary were given. The medication was adapted to age and comorbidities. Therefore, NSAIDs were avoided if possible. Analgesics were also gradually reduced as soon as possible. In THA, an attempt was made to discontinue the opioid after the first post-operative day. In very few cases, opioid analgesics had to be administered beyond this period. After four to six weeks (after rehabilitation), many patients were completely free of painkillers. After three months, painkillers were generally only taken for other medical issues (e.g., osteoarthritis of other joints, back pain, other chronic diseases), but no longer because of the hip operation.

### Limitations

This study has some limitations. The study is part of the ongoing SOG trial, and the analysis presented was not originally planned. For this reason, there is no control group for this evaluation. The improvement is not exclusively a benefit of the surgery. Postoperative care by the healthcare team, physiotherapy, and rehabilitation for functional exercise after joint replacement also contribute. The results were observed in this context. There is no differentiation between perioperative care with or without orthogeriatric co-management. This is a single-center study with corresponding limitations in the size and heterogeneity of the study population, as well as possible “center bias”. In addition, cognitive, visual, fine motor, and writing problems may all have led to distortions or confounding factors despite professional support in answering the questionnaires. A possible so-called “test subject motivation”, e.g., in the form of the effect of social desirability as a very elderly person, may also have led to a confounding factor. Similarly, accompanying persons (usually relatives) were often present when the questionnaires were collected. Although they were asked not to influence the answers to the questions by the study participants, their presence alone could theoretically have had an influence on the answers to the questions.

## 5. Conclusions

We showed a significant improvement in the functional outcome and health-related quality of life at four to six weeks and at three months following primary THA in the geriatric patient, using validated questionnaires. These results imply, that a geriatric patient benefits the same way from elective primary THA as a younger one. However, the preoperative comprehensive geriatric assessment with screening for risk factors is of utmost importance. Facing an aging population resulting from the demographic change, a lot of effort is needed to obtain more knowledge about geriatric patients receiving elective orthopedic surgery.

## Figures and Tables

**Table 1:** Demographic data of the 101 included patients.

Demographic Data (n = 101)												
Parameters	Mean ± SD						Range					
Age (years)	78.1 ± 4.9						70–89					
Sex (male:female)	36:65 (35.6%:64.4%)											
weight (kg)	78 ± 14.8						45–120					
height (cm)	165.4 ± 0.1						147–188					
Body mass index (BMI)	28.5 ± 5.1						18–48					
Obesity level (0–5)	0		1		2		3		4		5	
	1 (1%)		29 (26%)		36 (26%)		26 (19%)		6 (4%)		3 (2%)	
**Preoperative comprehensive geriatric assessment (CGA)**												
Level of care (0–3)	0			1			2			3		
	87 (86%)			4 (4%)			8 (8%)			2 (2%)		
Preexisting medication (number)	7.4 ± 4.0						1–21					
Comorbidities (number)	7.2 ± 3.3						1–22					
Charlson Comorbidity Index (CCI)	5.4 ± 2.0						3–14					
Mini Nutritional Risk Screening (NRS)	1–3						4					
	96 (95%)						5 (5%)					
Barthel Index preoperatively	93 ± 11.1						30–100					
Lawton and Brody Instrumental Activities of Daily Living Scale (IADL)	6.7 ± 1.8						0–8					
Fried Frailty Index	2.4 ± 1.2						0–4					
Mini Mental Status Test (MMST)	26.9 ± 2.8						10–30					
Geriatric Depression Scale (GDS)	3.3 ± 2.9						0–12					
Short Physical Performance Battery (SPPB)	7.1 ± 2.8						1–12					
**Peri- and postoperative parameters**												
Operated side (right:left)	50:51 (49.5%:50.5%)											
Duration of surgery (min)	64.2 ± 17.2						34–126					
Duration of hospitalization (d)	7.6 ± 2.4						7–29					
Prolongated hospitalization for medical reasons (d)	(n = 11) 0.5 ± 2.4						0–22					
Prolongated hospitalization for organizational reasons (d)	(n = 9) 0.2 ± 0.8						0–7					
Prolongated hospitalization for requirements of health insurance (d)	(n = 1) 0.02 ± 0.2						0–2					
Transfer to another hospital due to medical reasons (cases)	(n = 1) 0.01 ± 0.1						0–1					
Duration of re-hospitalization (d)	(n = 9) 1.1 ± 4.1						0–28					

**Table 2:** Results of the Western Ontario and McMaster Universities Osteoarthritis Index (WOMAC)—preoperative (t0), four to six weeks (t1) as well as three months (t2) postoperatively.

WOMAC (n = 101)			Statistical Difference (*p*-Value)		
		**(Mean ± SD)**	**t0 vs. T1**	t0 vs. t2	t1 vs. t2
Pain—walking in the plain (0–4)	PRE-OP	2.8 ± 0.8	**<0.001**	**<0.001**	**<0.001**
	4–6 w POST-OP	0.9 ± 0.8			
	3 m POST-OP	0.5 ± 0.7			
Pain—descending stairs (0–4)	PRE-OP	2.9 ± 0.9	**<0.001**	**<0.001**	**0.001**
	4–6 w POST-OP	0.9 ± 0.9			
	3 m POST-OP	0.6 ± 0.8			
Pain—sleeping (0–4)	PRE-OP	2.0 ± 1.2	**<0.001**	**<0.001**	**<0.001**
	4–6 w POST-OP	0.8 ± 0.7			
	3 m POST-OP	0.4 ± 0.7			
Pain—sitting/laying (0–4)	PRE-OP	2.1 ± 1.1	**<0.001**	**<0.001**	**0.001**
	4–6 w POST-OP	0.8 ± 0.7			
	3 m POST-OP	0.5 ± 0.8			
Pain—standing upright (0–4)	PRE-OP	2.4 ± 1.0	**<0.001**	**<0.001**	**<0.001**
	4–6 w POST-OP	0.8 ± 0.8			
	3 m POST-OP	0.5 ± 0.8			
Pain—need for medication (0–4)	PRE-OP	2.0 ± 1.4	0.99	**<0.001**	**<0.001**
	4–6 w POST-OP	2.0 ± 0.9			
	3 m POST-OP	0.5 ± 0.8			
Stiffness—morning (0–4)	PRE-OP	2.5 ± 1.1	**<0.001**	**<0.001**	**0.002**
	4–6 w POST-OP	1.2 ± 0.9			
	3 m POST-OP	0.9 ± 0.9			
Stiffness—during the course of day (0–4)	PRE-OP	2.5 ± 1.0	**<0.001**	**<0.001**	0.09
	4–6 w POST-OP	1.0 ± 0.8			
	3 m POST-OP	0.8 ± 0.8			
Problems—descending stairs (0–4)	PRE-OP	2.6 ± 1.1	**<0.001**	**<0.001**	**0.003**
	4–6 w POST-OP	1.0 ± 1.0			
	3 m POST-OP	0.6 ± 0.9			
Problems—climbing stairs (0–4)	PRE-OP	2.8 ± 0.9	**<0.001**	**<0.001**	**0.005**
	4–6 w POST-OP	1.1 ± 1.0			
	3 m POST-OP	0.7 ± 0.9			
Problems—getting up from chair (0–4)	PRE-OP	2.8 ± 1.0	**<0.001**	**<0.001**	**<0.001**
	4–6 w POST-OP	1.0 ± 0.9			
	3 m POST-OP	0.6 ± 0.8			
Problems—standing (0–4)	PRE-OP	2.3 ± 1.0	**<0.001**	**<0.001**	**<0.001**
	4–6 w POST-OP	1.0 ± 0.8			
	3 m POST-OP	0.5 ± 0.8			
Problems—bending over (0–4)	PRE-OP	2.8 ± 1.1	**<0.001**	**<0.001**	**<0.001**
	4–6 w POST-OP	1.8 ± 1.3			
	3 m POST-OP	1.1 ± 1.1			
Problems—walking in plain (0–4)	PRE-OP	2.5 ± 0.8	**<0.001**	**<0.001**	0.12
	4–6 w POST-OP	0.6 ± 0.7			
	3 m POST-OP	0.4 ± 0.7			
Problems—getting in/out of car (0–4)	PRE-OP	2.9 ± 1.0	**<0.001**	**<0.001**	**<0.001**
	4–6 w POST-OP	1.5 ± 1.0			
	3 m POST-OP	0.8 ± 0.9			
Problems—shopping (0–4)	PRE-OP	2.6 ± 1.0	**<0.001**	**<0.001**	**<0.001**
	4–6 w POST-OP	1.3 ± 1.2			
	3 m POST-OP	0.6 ± 0.9			
Problems—putting socks on (0–4)	PRE-OP	3.0 ± 1.1	**<0.001**	**<0.001**	**0.003**
	4–6 w POST-OP	1.7 ± 1.3			
	3 m POST-OP	1.2 ± 1.1			
Problems—getting up from bed (0–4)	PRE-OP	2.4 ± 1.0	**<0.001**	**<0.001**	**0.003**
	4–6 w POST-OP	1.0 ± 0.9			
	3 m POST-OP	0.7 ± 0.8			
Problems—putting socks off (0–4)	PRE-OP	2.8 ± 1.2	**<0.001**	**<0.001**	**0.004**
	4–6 w POST-OP	1.3 ± 1.2			
	3 m POST-OP	0.8 ± 1.0			
Problems—laying in bed (0–4)	PRE-OP	2.0 ± 1.0	**<0.001**	**<0.001**	**<0.001**
	4–6 w POST-OP	0.7 ± 0.8			
	3 m POST-OP	0.3 ± 0.7			
Problems—getting in/out of shower/bathtub (0–4)	PRE-OP	2.5 ± 1.4	**<0.001**	**<0.001**	**0.03**
	4–6 w POST-OP	0.9 ± 1.1			
	3 m POST-OP	0.6 ± 0.9			
Problems—sitting (0–4)	PRE-OP	1.9 ± 1.1	**<0.001**	**<0.001**	**0.03**
	4–6 w POST-OP	0.6 ± 0.7			
	3 m POST-OP	0.4 ± 0.6			
Problems—getting of toilet (0–4)	PRE-OP	2.3 ± 1.0	**<0.001**	**<0.001**	**0.02**
	4–6 w POST-OP	0.7 ± 0.8			
	3 m POST-OP	0.5 ± 0.7			
Problems—doing heavy housework (0–4)	PRE-OP	3.0 ± 1.0	**<0.001**	**<0.001**	**<0.001**
	4–6 w POST-OP	2.1 ± 1.3			
	3 m POST-OP	1.3 ± 1.2			
Problems—doing light housework (0–4)	PRE-OP	2.2 ± 0.9	**<0.001**	**<0.001**	**<0.001**
	4–6 w POST-OP	1.0 ± 1.0			
	3 m POST-OP	0.5 ± 0.9			
Total result WOMAC (0–100)	PRE-OP	62.6 ± 18.6	**<0.001**	**<0.001**	**<0.001**
	4–6 w POST-OP	27.1 ± 16.1			
	3 m POST-OP	16.2 ± 15.2			

**Table 3:** Results of the EQ-5D-5L—preoperative (t0), four to six weeks (t1) as well as three months (t2) postoperatively.

EQ-5D-5L (n = 101)			Statistical Difference (*p*-Value)		
		**(Mean ± SD)**	**t0 vs. t1**	**t0 vs. t2**	**t1 vs. t2**
Mobility (1–5)	PRE-OP	3.5 ± 0.8	**<0.001**	**<0.001**	0.99
	4–6 w POST-OP	1.7 ± 0.8			
	3 m POST-OP	1.7 ± 0.9			
Self-supply (1–5)	PRE-OP	2.1 ± 1.1	**0.005**	**<0.001**	0.06
	4–6 w POST-OP	1.7 ± 1.0			
	3 m POST-OP	1.5 ± 0.8			
General tasks (1–5)	PRE-OP	2.9 ± 1.2	**<0.001**	**<0.001**	**0.01**
	4–6 w POST-OP	2.0 ± 1.0			
	3 m POST-OP	1.7 ± 0.9			
Pain (1–5)	PRE-OP	3.7 ± 0.8	**<0.001**	**<0.001**	0.35
	4–6 w POST-OP	2.0 ± 0.9			
	3 m POST-OP	1.9 ± 0.9			
Anxiety (1–5)	PRE-OP	1.5 ± 0.8	**0.004**	**<0.001**	0.64
	4–6 w POST-OP	1.1 ± 0.3			
	3 m POST-OP	1.4 ± 0.7			
Total result EQ-5D-5L	PRE-OP	37.510 ± 855	**<0.001**	**<0.001**	0.99
	4–6 w POST-OP	19.363 ± 896			
	3 m POST-OP	18.729 ± 955			
Overall health (0–100)	PRE-OP	48.4 ± 21.1	**<0.001**	**<0.001**	0.54
	4–6 w POST-OP	68.7 ± 19.3			
	3 m POST-OP	72.8 ± 16.5			

## Data Availability

On request, data are available at the authors’ institution.
